# S244. CHARACTERIZING OUTCOMES OF CLINICAL HIGH-RISK NON-CONVERTERS USING GROUP-BASED TRAJECTORY MODELING

**DOI:** 10.1093/schbul/sby018.1031

**Published:** 2018-04-01

**Authors:** Dana Allswede, Tyrone Cannon, Jean Addington, Carrie Bearden, Kristin Cadenhead, Barbara Cornblatt, Daniel Mathalon, McGlashan Thomas, Diana Perkins, Larry Seidman, Ming Tsuang, Elaine Walker, Scott Woods

**Affiliations:** 1 Yale University; 2 University of Calgary; 3 University of California, Los Angeles; 4 University of California, San Diego; 5 The Zucker Hillside Hospital; 6 University of California, San Francisco; 7 University of North Carolina; 8 Harvard Medical School; 9 Emory University

## Abstract

**Background:**

The development of the clinical high-risk (CHR) prodromal criteria has facilitated advancement in understanding conversion to psychosis and has provided opportunities for early intervention and treatment for these individuals. However, the majority of CHR cases do not meet full criteria for conversion, yet continue to experience clinically significant symptoms and impairment in daily functioning. It is likely that many of these individuals would also benefit from additional intervention and treatment, but the outcomes and needs of these “non-converters” are not well characterized. Identifying common longitudinal patterns of symptoms and functioning of non-converters would support the identification of individuals who continue to require treatment and tailoring of services to their specific needs.

**Methods:**

We used group-based trajectory modeling to identify common longitudinal symptom and functioning trajectories among CHR cases (N=561) in the second phase of the North American Prodrome Longitudinal Study (NAPLS2). Covariant trajectories of symptoms (including positive, negative, disorganized, and general) and functioning (including role and social) were examined. Models were tested for replicability in an independent sample of CHR cases (N=291) from the first phase of NAPLS (NAPLS1).

**Results:**

We identified a subgroup of individuals who exhibited symptom remission and functioning within the normal range, as well as at least two additional subgroups that exhibited different patterns of ongoing, clinically significant symptoms and functional deficits.

**Discussion:**

We are currently investigating the validity of these subgroups by assessing their association with a variety of risk factors and biomarkers.

